# Plasma Extracellular Vesicle MicroRNA Analysis of Alzheimer’s Disease Reveals Dysfunction of a Neural Correlation Network

**DOI:** 10.34133/research.0114

**Published:** 2023-04-13

**Authors:** Yuzhe Sun, Zhen Hefu, Benchao Li, Wang Lifang, Song Zhijie, Li Zhou, Yan Deng, Liu Zhili, Jiahong Ding, Tao Li, Wenwei Zhang, Nie Chao, Shuang Rong

**Affiliations:** ^1^ Department of Nutrition and Food Hygiene, School of Public Health, Medical College, Wuhan University of Science and Technology, Wuhan, China.; ^2^ BGI-Shenzhen, Shenzhen, China.; ^3^ Shenzhen Key Laboratory of Neurogenomics, BGI-Shenzhen, Shenzhen 518120, China.; ^4^ College of Life Sciences, University of Chinese Academy of Sciences, Beijing 100049, China.

## Abstract

Small extracellular vesicle (sEV) is an emerging source of potential biomarkers of Alzheimer's disease (AD), but the role of microRNAs (miRNAs) in sEV is not well understood. In this study, we conducted a comprehensive analysis of sEV-derived miRNAs in AD using small RNA sequencing and coexpression network analysis. We examined a total of 158 samples, including 48 from AD patients, 48 from patients with mild cognitive impairment (MCI), and 62 from healthy controls. We identified an miRNA network module (M1) that was strongly linked to neural function and showed the strongest association with AD diagnosis and cognitive impairment. The expression of miRNAs in the module was decreased in both AD and MCI patients compared to controls. Conservation analysis revealed that M1 was highly preserved in the healthy control group but dysfunctional in the AD and MCI groups, suggesting that changes in the expression of miRNAs in this module may be an early response to cognitive decline prior to the appearance of AD pathology. We further validated the expression levels of the hub miRNAs in M1 in an independent population. The functional enrichment analysis showed that 4 hub miRNAs might interact with a GDF11-centered network and play a critical role in the neuropathology of AD. In summary, our study provides new insights into the role of sEV-derived miRNAs in AD and suggests that M1 miRNAs may serve as potential biomarkers for the early diagnosis and monitoring of AD.

## Introduction

Neurodegeneration-derived Alzheimer's disease (AD) is the second most prevalent affliction among elderly populations [1]. Due to the prolonged pathological progression of AD and its latency, the clinical presentation of AD is classified into 3 categories: cognitively normal, mild cognitive impairment (MCI), and full-blown AD [2]. MCI due to AD refers to an intermediate state between normal cognitive aging and symptomatic AD. It is characterized by memory loss, which is inconsistent with age but has not yet reached the clinical diagnosis standard of dementia [3–5]. Longitudinal cohort studies suggest that slowly progressing neuropathological changes, such as amyloid β (Aβ) deposition, begin in the early stages of MCI [6,7]. It is plausible that extending the early stages of MCI may delay the onset of AD pathology, underscoring the need to identify biomarkers of pathological and molecular changes during this stage [6]. However, early diagnosis of AD is challenging because neurodegenerative phenotypes are frequently accompanied by several cognitive disorders that are nearly indistinguishable from age-related cognitive decline [4,7]. Despite efforts to identify early-stage AD biomarkers, the most important clinical indicators remain Aβ and Tau [8,9].

Small extracellular vesicle (sEV), which contains proteins and RNAs that are transported between cells over distances that could trigger changes in gene expression and cellular function, exhibits a unique mode of cell communication [10–12]. As sEVs are capable of crossing the blood–brain barrier, they represent a valuable asset in the molecular diagnostics of neurological diseases [13]. In the central nervous system (CNS), 70% of microRNAs (miRNAs) are released from human brain cells and possibly regulate transcription of more than one-third of all genes [14,15]. Tissue-specific miRNAs can be isolated from enriched extracellular vesicles such as exosomes, the most studied subtype of EVs. In brain disorders, neuronal cells release sEVs that are enriched with miRNAs and other cellular components, which spread from the cerebrospinal fluid (CSF) into the peripheral blood system [16]. The presence of sEVs in the CSF, detectable in peripheral blood, makes them a promising pool of potential biomarker for neurodegenerative disease [17].

To date, several potential miRNA biomarkers for neurodegenerative disorders (NDs), including AD, Parkinson's disease, Huntington's disease, and other memory disorders, have been examined in blood, plasma, and serum by either quantitative polymerase chain reaction (qPCR) or small RNA (sRNA) sequencing, including miR-149-5p, miR-124-3p, miR-9-3p/5p, and miR-125b-5p [18–21]. However, the complexity of miRNA regulation presents challenges in fully understanding gene interaction networks. Weighted gene correlation network analysis (WGCNA) was developed to identify highly correlated expression patterns and calculate relationships between selected modules and external sample traits [22]. Gene expression-based network analysis has demonstrated its usefulness in the prognosis of various ND subtypes [23,24]. The identification of these disorders at an early stage through molecular characterization holds the potential to enhance the precision and prediction of diagnosis. Aberrantly expressed genes play a modular role in the progression of disease symptoms, and the deactivation and activation of coexpression networks may provide insight into the underlying mechanisms of NDs [23].

In this study, we aimed to explore the diagnostic potential of sEVs and their contained miRNA in the pathological classification of AD and MCI. To this end, we isolated sEVs from plasma of AD, MCI, and NC (normal control) and performed a comprehensive comparison of sEV miRNA expression between the 3 groups. Our results demonstrated the feasibility of using differentially expressed (DE) miRNAs as a diagnostic tool for differentiating between MCI and AD. Additionally, we performed WGCNA to uncover highly correlated patterns of gene expression and to assess the relationships between these patterns and the external traits of the samples. Our findings revealed a network that was strongly correlated with AD diagnosis and was conserved in NC but not in MCI and AD, implying its dysfunction in the latter 2 groups. Conversely, the majority of miRNA patterns were found to be similar between NC and MCI, suggesting that this network could serve as a biomarker of response to cognitive impairment. Tissue-specific analysis showed that most hub miRNAs in this network were expressed predominantly in the brain, providing evidence for the hypothesis that these miRNAs may originate from the CNS and serve as markers of posttranscriptional regulation in this organ. These results provide a basis for future studies aimed at uncovering the mechanisms underlying the progression of NDs and developing novel strategies for early diagnosis and intervention.

## Results

### Clinical and demographic of participants

**Fig. 1.**
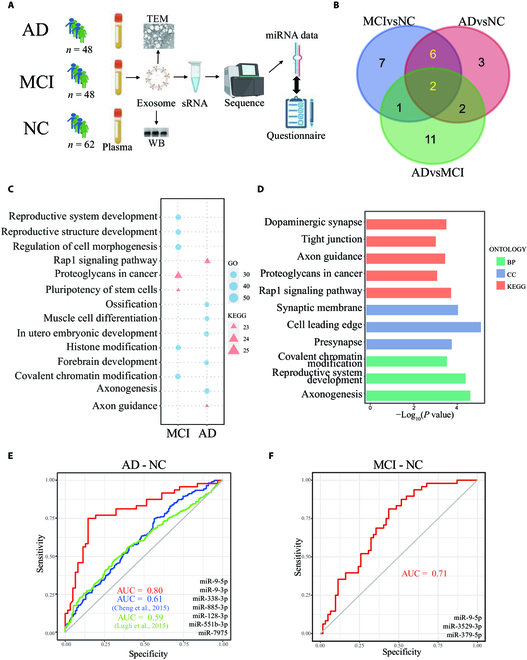
The plasma sEV miRNA profiles in AD, MCI, and NC participants. (A) Schematic diagram showing samples and analysis procedure of this study. The plasma samples were collected from 62 healthy volunteers, 48 MCI patients, and 48 AD patients. (B) Venn diagram of DE miRNAs in AD versus NC, MCI versus NC, and AD versus MCI comparisons. Yellow, expression levels of 8 miRNAs significantly changed in both AD and MCI. (C) GO and Kyoto Encyclopedia of Genes and Genomes (KEGG) enrichment of altered miRNAs. (D) GO and KEGG enrichment of 8 declined miRNAs in both MCI and AD. (E) The receiver operating characteristic (ROC) analysis shows that the AUC of AD-NC classifier is 0.80 in this study, and 0.61 and 0.59 in 2 published studies [31,32]. (F) ROC analysis shows that the AUC of MCI-NC classifier is 0.71.

To investigate the miRNA expression profile of AD patients, we collected 158 plasma samples from 48 AD cases, 48 sporadic MCI cases, and 62 NC cases (Fig. 1A). The diagnosis of AD and MCI was made according to the clinical diagnostic criteria set forth by the National Institute on Aging and Alzheimer's Association (NIA-AA) and Petersen's criteria, respectively [3,25,26]. To evaluate cognitive function, patients were subjected to various cognitive tests, including mini-mental state examination (MMSE), clinical dementia rating (CDR), Hachinski ischemic score (HIS), and geriatric depression scale (GDS). Detailed demography of patients is shown in Table.

**Table.** Clinical and demographic data of participants.

**Characteristics**	**AD patients (*n* = 48)**	**MCI patients (*n* = 48)**	**Controls (*n* = 62)**	***P* value**
Age, years (SD)	73.3 (7.4)	71.5 (5.5)	71.3 (5.6)	0.213
Male, *n* (%)	16 (33.3)	13 (27.1)	19 (30.6)	0.800
MMSE scores, mean (SD)	12.8 (4.0)	25.5 (4.0)	28.8 (2.0)	<0.001
MoCA scores, mean (SD)	NA	20.6 (3.0)	24.5 (4.6)	<0.001
BMI, mean (SD)	20.7 (3.2)	23.1 (3.1)	23.6 (3.1)	<0.001
Education (score 1/2/3)	47/1/0	8/27/13	30/18/14	<0.001
Marriage (score 1/2/3/4)	3/23/0/22	0/40/1/7	0/45/1/16	<0.001
Coronary heart disease	5	1	2	0.213
Hypertension	16	16	10	<0.05
Diabetes	1	4	5	0.392
Stroke	0	11	5	<0.001
Kidney disease	3	2	4	0.915
Tumor	0	1	1	1
Smoking (score 1/2/3)	12/0/36	6/4/38	4/4/54	<0.05
Alcohol (score 1/2/3)	7/0/41	7/3/38	9/5/48	0.377
Tea	2	17	18	<0.001
Entertainment	1	12	10	<0.01
Reading frequency	1	10	11	<0.01
Exercise (score 0/1/2/3)	4/0/6/38	4/36/5/3	2/43/13/4	<0.001
Communicate with neighbors (score 1/2/3)	32/4/12	24/20/4	35/23/4	<0.001
Communicate with children (score 0/1/2/3)	11/7/3/27	0/33/15/0	0/47/14/1	<0.001

Age, BMI, MoCA, and MMSE scores are depicted as mean (SD), and the *P* values are calculated by Kruskal–Wallis *H*-test (age, MoCA, and MMSE) and one-way ANOVA (BMI). Gender is depicted as a ratio of male to female, and the *P* values are calculated by Fisher’s exact test. Education, marriage, coronary heart disease, hypertension, diabetes, stroke, kidney disease, tumor, smoking, alcohol, tea, entertainment, reading frequency, exercise, communicate with neighbors, and communicate with children are depicted as the population counting, and the *P* values are calculated by Fisher’s exact test.

Demographic analysis showed that there were no significant differences in age or sex among the AD, MCI, and NC groups (Table). However, the MMSE scores were significantly lower in both the AD and MCI groups compared to the NC group, while the Montreal Cognitive Assessment (MoCA) scores were significantly lower in the MCI group compared to the NC group (Table and Table S1). These findings indicate a continuous decline in cognitive function across the 3 groups. Body mass index (BMI) was significantly lower in the AD group than in the MCI and NC groups. To gain a comprehensive understanding of the participants, disease history and lifestyle were collected via detailed initial health and demographic questionnaires. Education and marriage differed among 3 groups, which was consistent with the previous studies [27]. Furthermore, our findings suggested that preexisting conditions of hypertension and stroke might predispose individuals to developing AD. Lifestyle factors such as smoking, tea drinking, entertainment, reading frequency, exercise, and communication with neighbors and children varied significantly among the 3 groups (Table).

### Expression profiles of sEV miRNAs in AD and MCI patients

We isolated sEVs from plasma samples of AD, MCI, and NC groups, using a previously described precipitation method [17]. Transmission electron microscopy and Western blot were performed to confirm successfully isolated sEVs. Additionally, Brownian motion-based nanoparticle tracking analysis (NTA) was applied to measure distribution of nanoparticle size in the 3 groups (Fig. S1).

Following the extraction and sequencing of sRNA, we identified 2,026 miRNAs from 158 sRNA libraries (Fig. 1A and Table S2). Using R packages DESeq2 and EdgeR, 11 and 16 miRNAs were found to be significantly down-regulated in AD and MCI, respectively, compared to NC. MiR-6891-5p and miR-7975 were significantly up-regulated in AD compared to NC, and no up-regulated miRNA was observed in MCI, pointing toward a general decline of EV-derived miRNAs in both AD and MCI (Fig. 1B and Table S3). The down-regulation of miR-9-3p and miR-9-5p was also observed, which was consistent with previous research in brain tissues [28,29]. Interestingly, the expression levels of 14 miRNAs were significantly higher and 2 miRNAs were significantly lower in AD as compared to MCI (Table S3).

Target prediction and gene enrichment analysis showed that the DE miRNAs in AD were enriched in pathways related to axonogenesis, forebrain development, axon guidance, and Rap1 signaling, consistent with the widely observed dysfunction of synapses in AD patients (Fig. 1C) [30]. DE miRNAs in MCI were enriched in pathways related to reproductive system/structure development, regulation of cell morphogenesis, histone modification, and covalent chromatin modification (Fig. 1C). We also identified 8 miRNAs commonly suppressed in both AD and MCI, enriched in axon- and synapse-related functions (Fig. 1D).

Our study further examined the predictive powers of DE miRNAs as biomarkers, constructing 7-miRNA models of AD-NC and 3-miRNA models of MCI-NC, which showed area under the curve (AUC) values of 0.80 (*P* < 0.001) and 0.71 (*P* < 0.001), respectively. Besides, we downloaded sEV miRNA profiles of previous studies, and using the same miRNAs and criteria, AUC of the AD-NC model was 0.61 and 0.59 (Fig. 1E and F) [31,32]. However, when compared to a previous study (AUC = 0.91, *P* < 0.001) that combined plasma P-tau217, memory, executive function, and APOE, our results indicated that plasma sEV miRNA biomarkers were not more advantageous in AD diagnosis than traditional biomarkers [33].

### Construction of a consensus AD coexpression network

**Fig 2.**
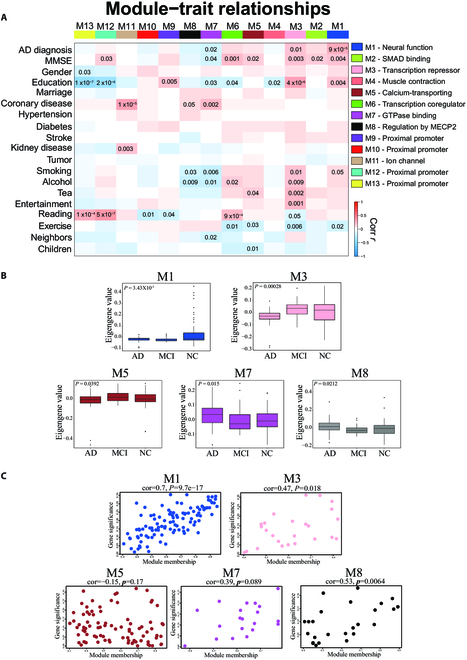
Co-expression network analysis of miRNA profiles in AD, MCI, and NC. (A) A correlation network consisting of 13 modules is generated from the top 1000 abundant miRNAs. Module eigengenes are correlated with AD diagnosis, cognitive function, and personal surveys. A 2-color heatmap shows the strength of the positive (red) or negative (blue) correlation, with *P* < 0.05 values provided. GO analysis of the miRNA targets within each module annotates the biological processes associated with the module. (B) Module eigengene values by case status for M1, M3, M5, M7, and M8 modules. Case status is from 158 participants. Differences in eigengene values are calculated by Kruskal–Wallis one-way analysis of variance (ANOVA). (C) A scatterplot of gene significance for AD diagnosis versus the module membership in the M1, M3, M5, M7, and M8 modules. Intramodular analysis of the genes found in M1, M3, M5, M7, and M8, which contains genes that have a high correlation with AD diagnosis. *P* value and correlation are on top of each box.

To further investigate the biological function of sEV miRNA, we used the WGCNA algorithm to generate a coexpression network from the 1,000 most abundant miRNAs. The resulting network consisted of 13 miRNA coexpression modules of similar expression patterns across the 158 cases analyzed (Fig. S2). The modules ranged in size from 202 miRNAs (M12, turquoise) to 12 miRNAs (M4, salmon). GO analysis of miRNA module members revealed putative functions of 13 modules, out of which 11 modules had a clear ontology, encompassing a diverse mix of biological processes and functions (Fig. 2A and Table S4). To assess whether a given coexpression module was related to either AD or MCI phenotypes, we correlated module eigengene values (the first principal component of the module miRNA expression level) to hallmarks of demography, personal health, and habits. We also correlated module eigengene values to cognitive function as assessed by the MMSE to capture module–AD/MCI relationships (Fig. 2A). We observed 3 modules that were significantly correlated with AD diagnosis and MMSE: M1 neural function, M3 transcription repressor, and M7 guanosine triphosphatase (GTPase) binding. The M1 neural function module exhibited the strongest AD trait correlations (AD diagnosis, *P* = 9 × 10^−5^; MMSE, *P* = 0.004), indicating that expression levels of M1 miRNAs might regulate AD neuropathology. Besides, M1 was correlated to education, smoking, and reading, which are important factors in AD development [34–36]. The M3 transcription repressor and M7 GTPase binding were correlated with education and numerous personal habits. Education level and reading behavior were associated with more than half of the modules (Fig. 2A). These findings suggested that biological regulation of M1, M3, and M7 might be altered during disease progression. It is also worth noting that 4 modules (M2 SMAD binding, M5 calcium transporting, M6 transcription coregulator, and M12 proximal promoter) were correlated with MMSE (*P* < 0.05), indicating that the regulatory functions of these modules are possibly affected by cognition decline.

To investigate the relationship between diagnostic classification and coexpression modules, we compared the module eigengene values by case status. Five modules exhibited significant differences among 3 diagnostic categories, including M1 neural function, M3 transcription repressor, M5 calcium transporting, M7 GTPase binding, and M8 regulation by MECP2 modules (Fig. 2B). Compared to NC, eigengene values of AD and MCI in M1 were significantly lower (*P* = 3.43 × 10^−5^), eigengene values of M3 and M5 decreased in AD but increased in MCI (*P* = 0.00028 and 0.0392, respectively), while eigengene values of M7 and M8 increased in AD but decreased in MCI (*P* = 0.015 and 0.0212, respectively) (Fig. 2B). An intramodular analysis of gene significance and module membership in the 5 modules was performed. AD diagnosis and module membership of M1, M3, and M8 exhibit significant correlation, and M1 was the most relevant module with AD (Fig. 2C). In summary, module eigengenes in AD when compared to NC were conversely altered in MCI, except M1. This suggested that M1 was the module especially related to AD, and miRNAs in M1 were potential biomarkers for AD staging.

The coexpression networks including M1, M3, M5, M7, and M8 contained 105, 25, 86, 20, and 25 miRNAs, respectively (Table S5). In order to determine the key miRNAs within each network, we calculated the membership of each miRNA (kME) and defined the miRNAs with the top 25% kME values in each coexpression module as “hub miRNAs.” Subsequently, we analyzed the beta diversity (sample dissimilarity) based on weighted distances, which was calculated using expression levels of hub miRNAs by case status. Unlike the previously discussed eigengene values, our results showed a significant decrease in diversity of AD and MCI compared to NC in modules M1, M3, M5, and M8. These findings suggest that the expression levels of hub miRNAs in the respective coexpression modules may have a shared pattern of change in the presence of AD or MCI (Fig. S3).

### The M1 model changed significantly in AD and MCI patients

In our study, we identified 26 hub miRNAs in the M1 network and discovered that 13 of these miRNAs were previously reported as AD markers (Table S6). Then, we queried the M1 hub miRNAs in the Human miRNA tissue atlas (https://ccb-web.cs.uni-saarland.de/tissueatlas/) and found that 24 of 26 M1 hub miRNAs were specifically expressed in brain tissues (Table S6), indicating a potential role for these miRNAs in nervous system function [37].

**Fig. 3.**
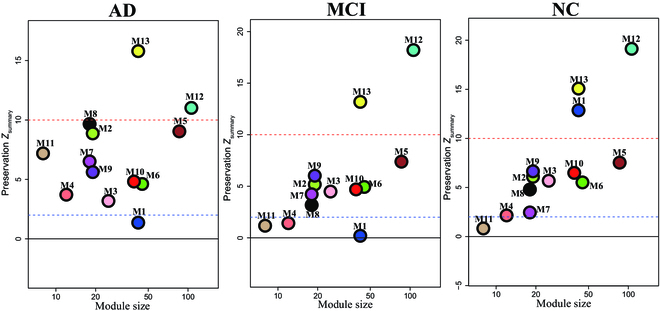
Preservation of miRNA network modules in 3 groups. Coexpression network preservation analysis in WGCNA R package. The dashed blue line indicates a *Z*_summary_ score of 2 or FDR *q* value < 0.05, above which module preservation is considered statistically significant. The dashed red line indicates a *Z*_summary_ score of 10 or FDR *q* value ~1 × 10^−23^.

To determine the status of the M1 network in AD, MCI, and NC, we evaluated the preservation of the model for each group (Fig. 3). We found that 12 of 13 network modules were preserved in AD and NC, and 10 of 13 modules were preserved in MCI. The preservation *Z*_summary_ values of 13 modules in MCI and NC were relatively close but changed greatly in AD, indicating that the miRNA coexpression networks did not change greatly in MCI, but rather in AD. Remarkably, M1 neural function was highly preserved in NC (*Z*_summary_ = 12.9) but not preserved in MCI (*Z*_summary_ = 0.2) and AD (*Z*_summary_ = 1.3), suggesting that the dysfunction of M1 miRNA network may occur at the MCI stage prior to the onset of AD. Additionally, M12 and M13 proximal promoters were highly preserved (*Z*_summary_ > 10) in all 3 groups, potentially regulating housekeeping functions that do not change during cognitive decline (Fig. 3).

### Association of sEV-derived M1 miRNAs with plasma proteins

**Fig. 4.**
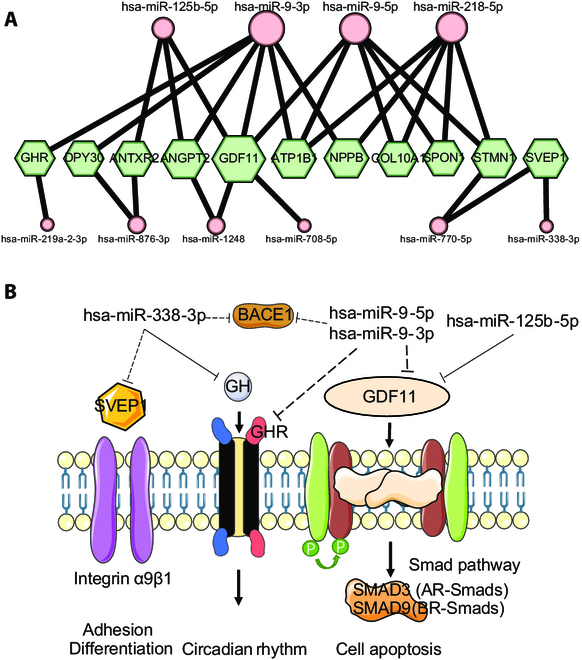
Association of M1 miRNA and plasma proteins. (A) Target prediction of M1 miRNA and 38 plasma proteins, which were associated with incident dementia. (B) Crosstalk between key miRNAs and molecular components of AD-related pathways.

We examined the relationship between M1 hub miRNAs and 38 plasma proteins that were reported associated with incident dementia [38]. By using a comprehensive approach that included target predictions from 8 different databases, we obtained 49 pairs of correspondences in at least 2 databases (Table S7). After removing the proteins that were only linked to one miRNA, the association of 11 genes with 10 M1 hub miRNAs was retained. Our analysis revealed that hsa-miR-125b-5p, hsa-miR-9-3p, hsa-miR-9-5p, and hsa-miR-218-5p emerged as key miRNAs, with growth differentiation factor 11 (GDF11) being the most highly connected gene (Fig. 4A). We also observed that the immunologically relevant cellular adhesion protein, Sushi, von Willebrand factor type A, epidermal growth factor, and pentraxin domain-containing protein 1 (SVEP1), which has been implicated in brain atrophy and Alzheimer's pathology, was targeted by hsa-miR-338-3p and involved in the transforming growth factor-β (TGF-β) signaling pathway. These findings suggest that the M1 miRNAs may contribute to Alzheimer's pathology through their involvement in a GDF11-centered network (Fig. 4B).

### Validation of M1 miRNAs by quantitative reverse transcription PCR

**Fig. 5.**
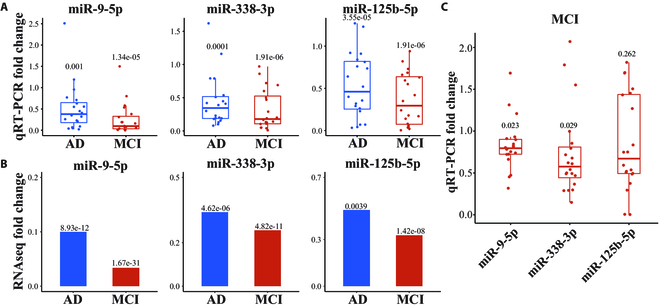
Quantitative RT-PCR validation for miR-9-5p, miR-338-3p, and miR-125b-5p. (A) Box plots represent relative expression of miR-9-5p, miR-338-3p, and miR-125b-5p in MCI and AD samples via qRT-PCR. The data points represent relative gene expression values normalized to the expression of miR-186-5p using the ddCt method. Thirty AD, 30 MCI, and 30 NC samples are used for qRT-PCR experiments. *P* values of *t* test are represented above the boxes. (B) Bar plots represent relative expression of miR-9-5p, miR-338-3p, and miR-125b-5p in MCI and AD samples in small RNA-seq data. *P* values of DE miRNAs are represented above the bars. (C) Quantitative RT-PCR validation for miR-9-5p, miR-338-3p, and miR-125b-5p in another 20 MCI and 20 NC community-based samples. Box plots represented relative expression of miR-9-5p, miR-338-3p, and miR-125b-5p of MCI/NC. *P* values of *t* test are represented above the boxes.

To validate the sRNA sequencing analysis, the abundance of selected miRNAs (miR-9-5p, miR338-3p, and miR-125b-5p) was validated by quantitative reverse transcription PCR (qRT-PCR) assay (Fig. 5A). We used 60 samples in the experiment, 20 AD, 20 MCI, and 20 NC samples. The qRT-PCR validation results of 3 miRNA expression levels corroborated those of the sequencing analyses (Fig. 5B). To reinforce the validity of the findings, the miRNA expression levels were also measured in a separate set of 40 samples (20 MCI and 20 NC). There were no significant differences in age, BMI, education, marriage, hypertension, diabetes, smoking, or alcohol consumption between MCI and NC samples. The MMSE scores of the MCI group were significantly lower than the NC group (Table S8). The MCI group had lower levels of miR-9-5p and miR-338-3p expressions than the NC group, consistent with our qRT-PCR results (Fig. 5C). The MCI group had insignificantly lower expression levels of miR-125b-5p than the NC group (Fig. 5C). These results provide additional support for the reliability of the sequencing analysis.

## Discussion

Aβ and Tau proteins have been widely studied as biomarkers for AD, and they have shown potential for the early diagnosis and monitoring of the disease. Expression of miRNA and accumulation of Aβ and Tau are different components of the biology of AD, and their potential for clinical diagnosis is also different. Growing evidence suggests that miRNAs contribute to the onset and development of NDs through synaptic plasticity and various signaling pathways [39–42]. MiRNAs are enveloped in sEVs and released from cells to the environment or circulation system, which can be recruited by cells at a distance or in another tissue where they can affect gene expression. However, enrichment of miRNA varies widely in plasma, blood immune cells, CSF, and brain tissue, and the biological functions of miRNA in sEVs of peripheral blood remain elusive [43,44].

In this study, we measured expression levels of sRNA from plasma sEVs in AD, MCI, and NC groups. The miRNA coexpression networks were constructed, and M1 neural function was presumed dysfunctional in AD and MCI (Fig. 3). Of the M1 hub miRNAs, both hsa-miR-9-5p and hsa-miR-9-3p are proved to be enriched in the CNS and regulate essential neuronal processes such as neuronal differentiation and synaptic plasticity [45–47]. In particular, as the potential target of miR-9-5p and miR-9-3p, GDF11 is a member of the TGF-β superfamily and is reportedly beneficial in preventing age-related degeneration in CNS, enhancing cognitive function, and stimulating tissue regeneration [48,49]. Besides hsa-miR-9-5p and has-miR-9-3p, hsa-miR-125b-5p negatively regulates *GDF11* expression, which triggers canonical signal transduction mediated by R-SMAD proteins (Fig. 4B). A previous study has indicated that a reduction in hsa-miR-125b-5p levels leads to an increase in *GDF11* expression and attenuates neuronal injury [50]. This supported the hypothesis that miRNAs in M1 may be involved in the underlying mechanisms of AD.

In the intricate dance of neural biology, *Beta-secretase 1* (*BACE1*) steps to the forefront as the leading performer, responsible for the creation of Aβ peptides [51]. Yet, recent studies have uncovered a perturbation in this delicate balance, and the decreased expression levels of hsa-miR-9-5p, hsa-miR-9-3p, and hsa-miR-338-3p lead to Aβ42 increase by targeting *BACE1* and exacerbating the disease [52,53]. Besides *BACE1*, hsa-miR-338-3p also targets *growth hormone* (*GH*) [54], whose receptor (GHR) was targeted by hsa-miR-9-3p and hsa-miR-9-5p, indicating that M1 miRNAs might be involved in the GH-related circadian rhythm (Fig. 4). SVEP1 is a newly reported plasma biomarker, high levels of which present a causal relationship with AD [38]. Combining hsa-miR-338-3p’s regulatory relation with *BACE1* and *GH* and its putative target *SVEP1*, hsa-miR-338-3p plays an essential role in the development of neuropathology in AD.

This study has some inherent limitations. First, the number of AD samples is relatively small owing to its low incidence. Another reason is that the period of AD diagnosis is usually more extended, and AD lacks effective treatment methods as well, which will affect patients' enthusiasm for participating in this study. Second, this community-based case–control study precluded us from making any temporal association between the miRNA expression and the AD diagnosis or cognitive decline. Next, the significance of M1 comes from the relationship between coexpression indices of interaction profiles within networks and clinical data of the AD patients. This relationship mainly indicates the biological mechanism of the network and the disease, rather than diagnostic power. Although we have validated our findings in another independent MCI and NC population, we cannot conduct an effective assessment of AD early diagnostic biomarkers from miRNA expression, and we would like to measure the miRNA expression by constructing a longitudinal cohort in the future.

In summary, we found a completely altered network preservation pattern in the AD group, corroborating the clinical pathology of AD cognitive impairment and MMSE score (Table). Our findings, backed by clinical pathology and cognitive impairment scores, paint a picture of a disrupted network and a dysfunction in the M1 neural function. The discovery of eight miRNAs with decreased expression, all found within the M1 module, and confirmed through tissue-specific analysis, provides further evidence of a breakdown in the delicate balance of posttranscriptional regulation. The M1 module could emerge as a potential source of biomarkers for the early detection of AD. Our investigation represents a critical step toward understanding the intricacies of the fundamental mechanisms of AD.

## Materials and Methods

### Participant enrollment

In this study, 158 individuals were enrolled, including 48 AD patients, 48 MCI patients, and 62 NC volunteers. Participants were from communities of Wuhan, Huangshi, and Jingmen Cities in Hubei province, China. Cases were frequency-matched by age and sex. Cognitive tests performed were MMSE, MoCA, CDR, HIS, and GDS. The diagnosis of AD was based on the criteria of the NIA-AA [25]. Briefly, the AD was diagnosed in clinical settings with the following criteria: (a) meeting the criteria for dementia, (b) excluding vascular dementia by distinguishing cerebrovascular diseases by computed tomography scans of the brain, (c) excluding the patients with prominent features of Lewy body dementia or frontotemporal dementia, and (d) excluding the active neurological diseases or other medication-induced cognitive disorders. The diagnosis of MCI was based on the Petersen's criteria from previous studies [3,26]. All participants' enrollment was approved by the Institutional Review Board of BGI (BG-IRB 20149) and the Medical Ethics Committee of Wuhan University of Science and Technology (No. 049). The standardized questionnaire was used to obtain demographic characteristics, history of diseases, and lifestyle factors. Written consent was obtained from all subjects.

### sEV isolation and characterization

To isolate sEVs from plasma, the commercial kit System Biosciences ExoQuick Kit was used in accordance with the manufacturer's instructions [12]. Briefly, plasma samples were first thawed and mixed by vortexing. Subsequently, 50 μl of plasma sample was subjected to centrifugation at 3,000*g* for 15 min to separate cells and cellular debris from the supernatant. The supernatant was then transferred to a sterile vessel, mixed with 63 μl of ExoQuick Precipitation Solution (63 μl), and refrigerated for 30 min. After refrigeration, the mixture was centrifuged at 1,500*g* for 30 min followed by a second centrifugation at 1,500g for 5 min to remove any residual ExoQuick solution. The resulting pellet was then resuspended in 25 μl of nuclease-free water.

### sRNA extraction and sequencing

sRNA was extracted from the resuspended sEV solution with the miRNeasy Serum/Plasma Kit (catalog no. 217084, QIAGEN, Germany) as per the manufacturer's instructions. Using Agilent's small RNA kit, sRNA samples were subsequently analyzed on a Bioanalyzer 2100 system (Agilent, Santa Clara, USA) to determine RNA integrity numbers (RINs). The concentration of sRNA was measured using NanoDrop 2000 (Thermo Scientific). sRNA sequencing libraries were prepared from total sEV sRNA with the MGIEasy Small RNA Library Prep Kit (MGI, Shenzhen, China). Pooled libraries were loaded and sequenced on a BGISEQ-500 platform (BGI), and more than 2.0 GB of reads were obtained from each library. Generated sequenced reads were deposited into the CNGB Sequence Archive (CNSA) of China National GeneBank DataBase (CNGBdb) under accession number CNP0001975 [55,56].

### sRNA data analysis

We generated an average of 2.2 Gb raw data per library from sRNA sequencing. Raw reads were processed by trimming adaptor sequences and then culling low-quality reads using SOAPnuke v1.5.0 (with the setting -Q 2 -q -c 0). High-quality reads that ranged from 70 to 500 Mb per sample were mapped against the reference genome (hg19) using Bowtie 2 (with the setting -q -L 16 -p 6 --phred64 --rdg 1,10 --rfg 1,10) [57] with allowance for only one mismatch. The matched reads were aligned to mature miRNAs in miRbase version 20 [58], and miRNA counts were calculated using perfectly matched reads. The remaining reads were mapped to the Rfam database [59] to predict novel miRNAs by identification and removal of protein-coding genes, transfer RNA (tRNA), and ribosomal RNA (rRNA). We used miRDeep2 to predict the novel miRNA from the retained reads [60].

Analysis of DE miRNA was performed using EdgeR and DESeq2 [61,62]. The thresholds for significant miRNA expression changes were >±1-fold (log_2_) and false discovery rate (FDR) ≤ 0.05, and *P* values were corrected to FDR using the Benjamin and Hochberg (BH) method. R package MultimiR was used to predict the targets of miRNA [63]. Gene ontology (GO) analysis was performed on putative target genes using clusterProfiler [64]. DE miRNAs were selected to calculate the diagnostic power. R package Caret was used to combine the predictive powers of DE miRNAs. Logistic regression with internal 10-fold cross-validation was used to develop the model. The AUC was calculated to assess the model's power by R package pROC.

### Weighted gene correlation network analysis

The coexpression network analysis was performed using the R package WGCNA [22]. To construct a weighted coexpression network, expression levels of 158 sRNA-seq samples were used, including AD, MCI, and NC groups. The WGCNA network function was used with the following settings: soft threshold power β = 5, deepSplit = 4, minimum module size of 12, merge cut height of 0.07. Pearson correlations between every miRNA and module eigengene were performed. After the initial network construction, 13 modules consisting of 12 to 202 miRNAs were detected. The first principal component (eigengene value) was calculated and considered as representative of each module. We discarded the gray module, which cannot be merged into any other modules, and then correlated the eigengene values of modules with phenotype traits shared by AD, MCI, and NC samples using the function corPvalueStudent() of the Pearson method. MiRNAs with the highest eigengene values were selected and predicted the targets, and functional enrichment analysis was performed using Metascape. Based on the GO annotation of miRNA targets, we then summarized the potential function of each network. Using the above networks as the template, *Z*_summary_ composite preservation scores were calculated for each target group, with 500 permutations. The modulePreservation() function in the WGCNA package was used.

### Real-time qRT-PCR

In this study, 60 samples were selected out of 158 for qRT-PCR validation (20 samples from each of the 3 groups). The sEV miRNA was converted into cDNA using the TaqMan Advanced miRNA cDNA Synthesis Kit (Applied Biosystems, #A28007), and qRT-PCR was performed on a StepOnePlus Real-Time PCR System using miRNA assays in a 96-well format (TaqMan microRNA assays, 20×, Applied Biosystems, #A25576). Has-miR-186-5p was used as an endogenous control. Data were tested for normality using the Shapiro test. Additionally, expression levels of miR-9-5p, miR338-3p, and miR-125b-5p were assessed in an independent set of samples composed of 20 MCI and 20 NC samples.
