# Supplementary material for: Plasma Extracellular Vesicle MicroRNA Analysis of Alzheimer’s Disease Reveals Dysfunction of a Neural Correlation Network
Source: Research (Wash D C). 2023 Apr 13;6:0114. doi: 10.34133/research.0114 (PMC10202186; doi:10.34133/research.0114)
Supplement: Supplementary 1 — Figs. S1 to S3 Tables S1 to S8 [file research.0114.f1.zip › Table S6. Hub miRNAs in M1 module.docx]

| **Table S6. Hub miRNAs in M1 module.** | | | |  |  | |  | |  | |
| --- | --- | --- | --- | --- | --- | --- | --- | --- | --- | --- |
| miRNA | Log (AD/NC) | *p_adjust_* | Log (MCI/NC) | *p*_adjust_ | | AD marker | | Tissue-specific | |  |
| miR-137 | -8.73 | 0.00 | -8.95 | 0.00 | | - | | Brain, Brain (grey matter) | |  |
| miR-219a-2-3p | -8.22 | 0.00 | -7.97 | 0.00 | | - | | Brain (white matter), Brain | |  |
| miR-149-5p | -5.55 | 0.00 | -6.19 | 0.00 | | + | | Brain, Brain(cerebellum)^1^ | |  |
| miR-138-5p | -4.55 | 0.00 | -4.81 | 0.00 | | + | | Brain (nucleus caudatus), Brain (cerebral cortex)^2^ | |  |
| miR-124-3p | -4.29 | 0.00 | -5.36 | 0.00 | | + | | Brain(cerebellum), Brain^3^ | |  |
| miR-219b-5p | -3.94 | 0.00 | -3.54 | 0.00 | | + | | NA^4^ | |  |
| miR-488-3p | -3.79 | 0.00 | -5.40 | 0.00 | | - | | Brain | |  |
| miR-219a-5p | -3.44 | 0.00 | -3.55 | 0.00 | | + | | Brain (white matter), Brain^5^ | |  |
| miR-9-5p | -3.33 | 0.00 | -4.89 | 0.00 | | + | | Brain (cerebellum), Brain^6^ | |  |
| miR-153-3p | -2.98 | 0.00 | -3.45 | 0.00 | | + | | Brain^6^ | |  |
| miR-383-5p | -2.61 | NA | -3.65 | 0.00 | | + | | Brain (cerebellum), Brain^7^ | |  |
| miR-708-5p | -2.61 | 0.00 | -2.99 | 0.00 | | - | | Colon, Myocardium | |  |
| miR-876-3p | -2.05 | NA | -1.29 | 0.30 | | - | | Brain | |  |
| miR-577 | -2.05 | NA | -4.22 | 0.00 | | - | | Brain (white matter), Spinal cord | |  |
| miR-129-1-3p | -2.00 | 0.05 | -3.74 | 0.00 | | - | | Brain, Brain (cerebral cortex) | |  |
| miR-129-2-3p | -1.99 | 0.00 | -2.42 | 0.00 | | - | | Brain, Brain (nucleus caudatus) | |  |
| miR-218-5p | -1.90 | 0.00 | -2.04 | 0.00 | | - | | Brain (cerebellum), Brain | |  |
| miR-376a-5p | -1.78 | NA | -2.86 | 0.00 | | - | | Brain | |  |
| miR-132-5p | -1.78 | NA | -3.16 | 0.02 | | + | | Brain, Brain (pituitary gland)^5^ | |  |
| miR-338-3p | -1.56 | 0.00 | -1.97 | 0.00 | | + | | Brain, Brain (white matter)^8^ | |  |
| miR-1248 | -1.30 | NA | -3.04 | 0.09 | | + | | Brain, Pancreas^9^ | |  |
| miR-9-3p | -1.22 | 0.00 | -2.43 | 0.00 | | + | | Brain, Brain (cerebellum)^6^ | |  |
| miR-125b-5p | -1.03 | 0.00 | -1.61 | 0.00 | | + | | Brain, Spinal cord^10^ | |  |
| miR-411-5p | -1.02 | 0.01 | -1.66 | 0.00 | | - | | Brain, Brain (cerebellum) | |  |
| miR-124-5p | -0.35 | NA | -2.23 | NA | | - | | Brain, Brain (cerebellum) | |  |
| miR-770-5p | 0.33 | NA | -1.66 | NA | | - | | Brain (nucleus caudatus), Brain (cerebellum) | |  |

Underline represents the miRNA significantly declined in AD and MCI.

+/- represents whether the corresponding miRNA is a reported AD biomarker

1 Chen, F. *et al.* miR-149-5p inhibition reduces Alzheimer's disease beta-amyloid generation in 293/APPsw cells by upregulating H4K16ac via KAT8. *Exp Ther Med* **20**, 88, doi:10.3892/etm.2020.9216 (2020).

2 Feng, X. *et al.* LncRNA 4344 promotes NLRP3-related neuroinflammation and cognitive impairment by targeting miR-138-5p. *Brain Behav Immun* **98**, 283-298, doi:10.1016/j.bbi.2021.08.230 (2021).

3 Zhou, Y., Deng, J., Chu, X., Zhao, Y. & Guo, Y. Role of Post-Transcriptional Control of Calpain by miR-124-3p in the Development of Alzheimer's Disease. *J Alzheimers Dis* **67**, 571-581, doi:10.3233/JAD-181053 (2019).

4 Li, J., Chen, W., Yi, Y. & Tong, Q. miR-219-5p inhibits tau phosphorylation by targeting TTBK1 and GSK-3beta in Alzheimer's disease. *J Cell Biochem* **120**, 9936-9946, doi:10.1002/jcb.28276 (2019).

5 Cha, D. J. *et al.* miR-212 and miR-132 Are Downregulated in Neurally Derived Plasma Exosomes of Alzheimer's Patients. *Front Neurosci* **13**, 1208, doi:10.3389/fnins.2019.01208 (2019).

6 Gupta, P. *et al.* miRNAs in Alzheimer Disease - A Therapeutic Perspective. *Curr Alzheimer Res* **14**, 1198-1206, doi:10.2174/1567205014666170829101016 (2017).

7 Zhou, Q., Luo, L., Wang, X. & Li, X. Relationship between single nucleotide polymorphisms in the 3'UTR of amyloid precursor protein and risk of Alzheimer's disease and its mechanism. *Biosci Rep* **39**, doi:10.1042/BSR20182485 (2019).

8 De Felice, B. *et al.* miR-338-3p is over-expressed in blood, CFS, serum and spinal cord from sporadic amyotrophic lateral sclerosis patients. *Neurogenetics* **15**, 243-253, doi:10.1007/s10048-014-0420-2 (2014).

9 Liu, Q. Y. *et al.* Identification of microRNAs involved in Alzheimer's progression using a rabbit model of the disease. *Am J Neurodegener Dis* **3**, 33-44 (2014).

10 Lugli, G. *et al.* Plasma Exosomal miRNAs in Persons with and without Alzheimer Disease: Altered Expression and Prospects for Biomarkers. *PLoS One* **10**, e0139233, doi:10.1371/journal.pone.0139233 (2015).
