# Supplementary material for: Plasma Extracellular Vesicle MicroRNA Analysis of Alzheimer’s Disease Reveals Dysfunction of a Neural Correlation Network
Source: Research (Wash D C). 2023 Apr 13;6:0114. doi: 10.34133/research.0114 (PMC10202186; doi:10.34133/research.0114)
Supplement: Supplementary 1 — Figs. S1 to S3 Tables S1 to S8 [file research.0114.f1.zip › Table S8. Demographic data of patients with MCI and NC.docx]

| **Table S8.** **Demographic data of patients with MCI and NC^a^.** | | | |
| --- | --- | --- | --- |
| **Characteristics** | **MCI (n=20)** | **NC (n=20)** | ***p*-value** |
| **Age, year (SD)** | 70.0(4.9) | 70.4(4.5) | 0.762 |
| **Male, n (%)** | 6(30.0) | 6(30.0) | 1.000 |
| **BMI, mean (SD)** | 24.8(3.6) | 23.7(3.4) | 0.293 |
| **MMSE scores, mean (SD)** | 22.2 (3.4) | 28.0 (1.5) | < 0.001 |
| **Education (score 1/2/3/4)** | 6/8/5/1 | 5/6/7/2 | 0.826 |
| **Married, n (%)** | 14 (70.0) | 15 (75.0) | 0.723 |
| **Hypertension, n (%)** | 9 (45.0) | 11 (55.0) | 0.527 |
| **Diabetes, n (%)** | 3 (15.0) | 3 (15.0) | 1.000 |
| **Smoking, n (%)** | 5 (25.0) | 3 (15.0) | 0.695 |
| **Alcohol use, n (%)** | 2 (10.0) | 5 (25.0) | 0.408 |

**^a^** MCI, mild cognitive impairment; NC, normal control; BMI, body mass index; MMSE, mini-mental state examination. Age, BMI, and MMSE scores are depicted as mean (SD), and the *p-values* are calculated by one-way ANOVA. Male, education, marriage, hypertension, diabetes, smoking, and alcohol are depicted as number of cases (%). The *p-values* for education, diabetes, smoking, and alcohol use are calculated by Fisher’s Exact Test. The *p-values* for sex, marriage, and hypertension are calculated by Pearson χ^2^. Education (score 1/2/3/4) are corresponding to the education level of illiteracy, primary school, junior high school, and high school and above.
