# Supplementary figures and images for: Plasma Extracellular Vesicle MicroRNA Analysis of Alzheimer’s Disease Reveals Dysfunction of a Neural Correlation Network
Source: Research (Wash D C). 2023 Apr 13;6:0114. doi: 10.34133/research.0114 (PMC10202186; doi:10.34133/research.0114)

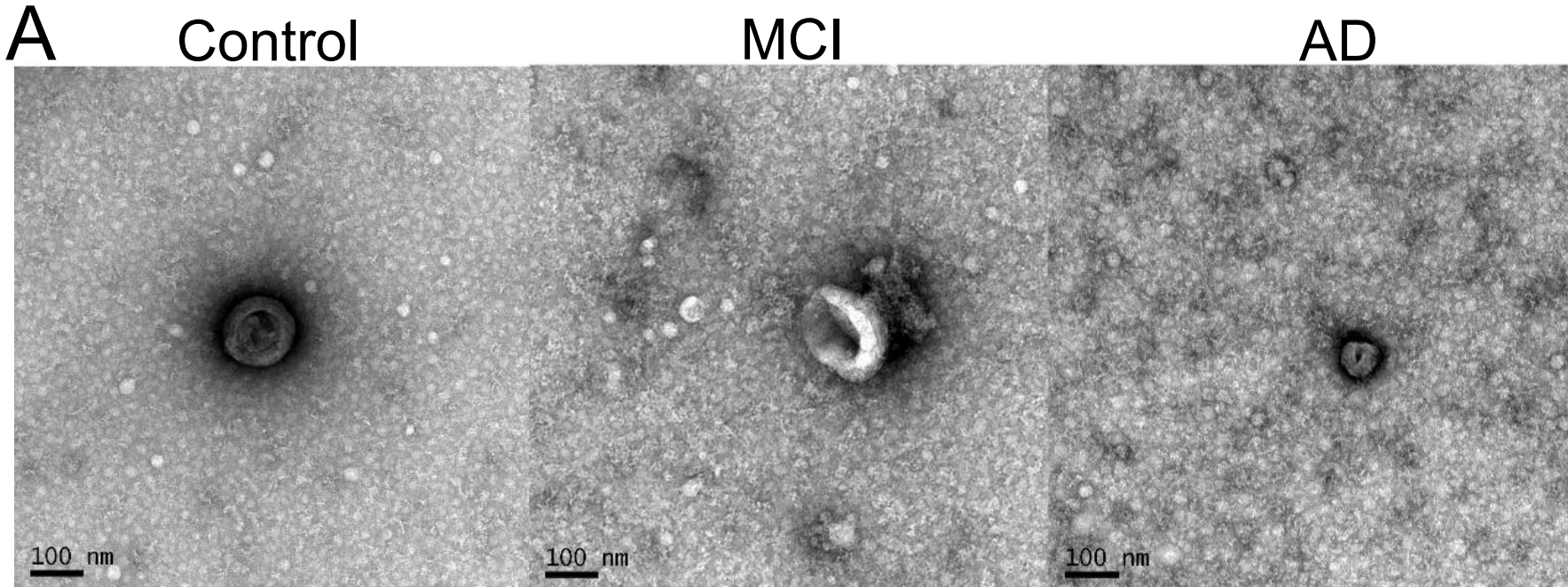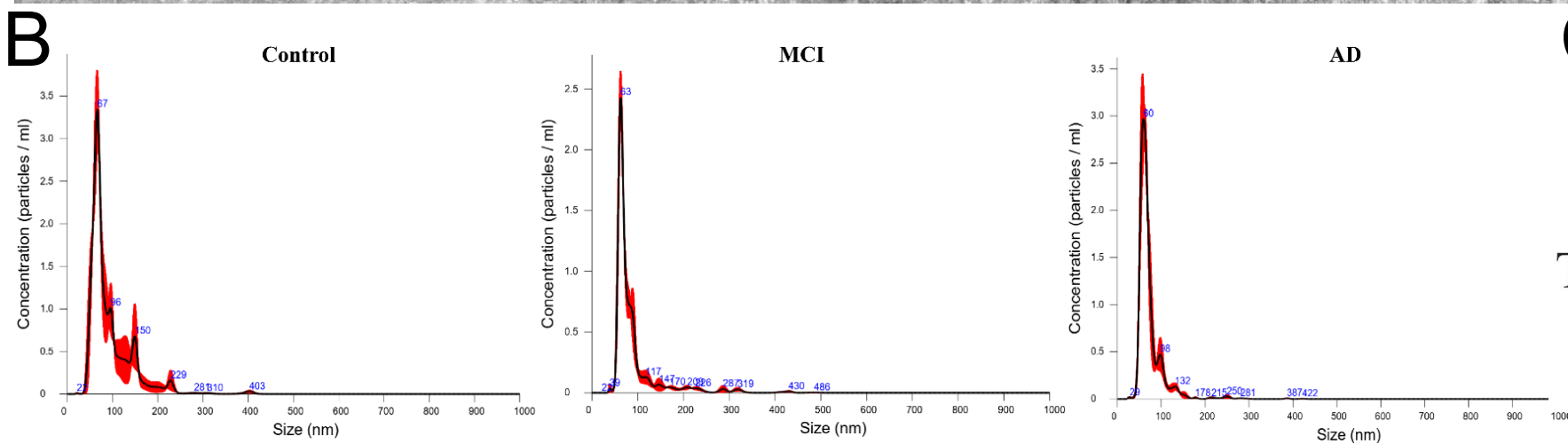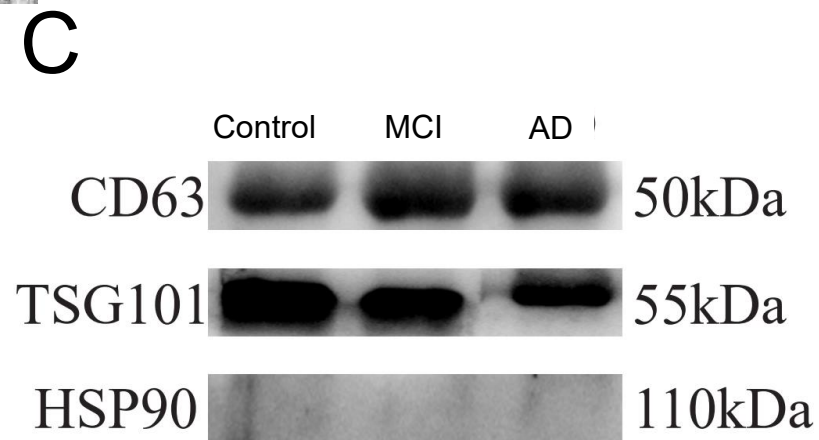

Supplement: Supplementary 1 — Figs. S1 to S3 Tables S1 to S8 [file research.0114.f1.zip › Figure S1. Validation of isolated small vesicle.pdf]

# Cluster Dendrogram

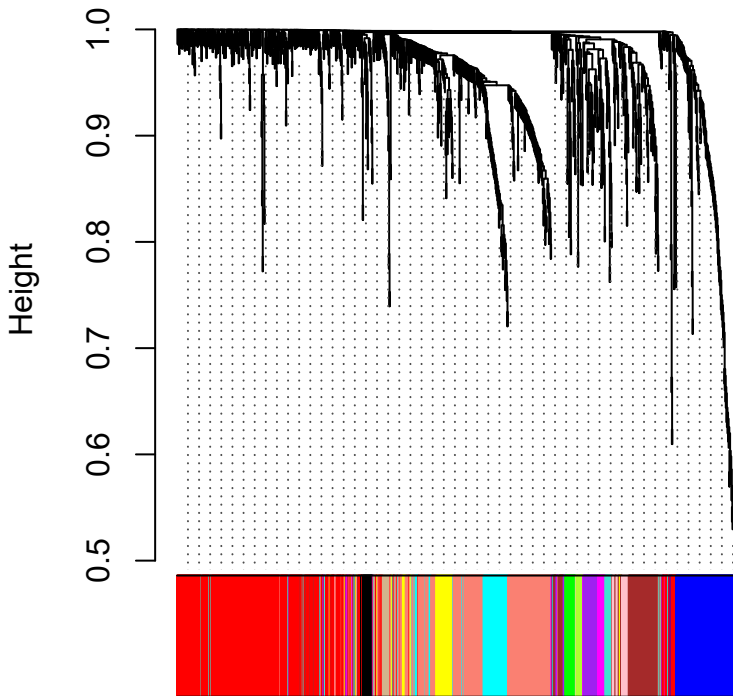

Supplement: Supplementary 1 — Figs. S1 to S3 Tables S1 to S8 [file research.0114.f1.zip › Figure S2. Clustering dendrogram of miRNAs.pdf]

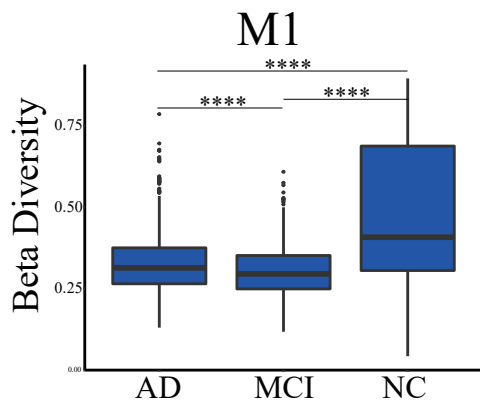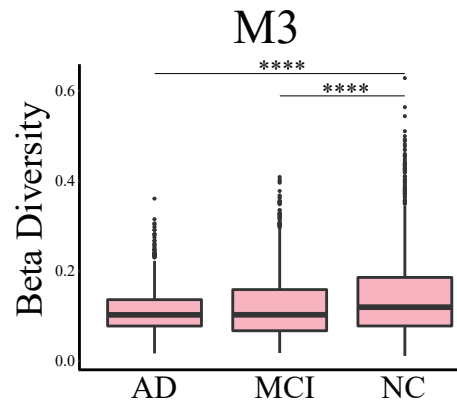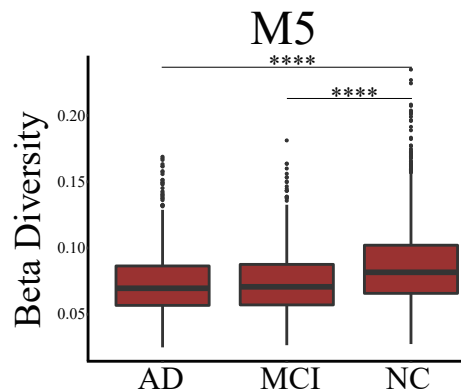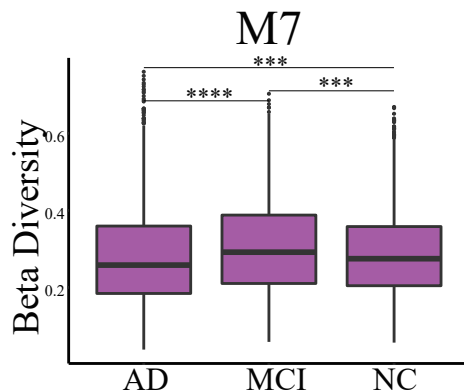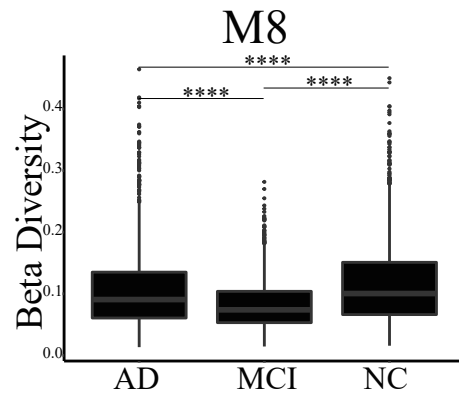

Supplement: Supplementary 1 — Figs. S1 to S3 Tables S1 to S8 [file research.0114.f1.zip › Figure S3. Beta diversity.pdf]
